# Speckle Tracking and Transthyretin Amyloid Cardiomyopathy

**DOI:** 10.5935/abc.20160191

**Published:** 2017-01

**Authors:** Alexandre Marins Rocha, Suzane Garcia Ferreira, Marcelo Souto Nacif, Mario Luiz Ribeiro, Marcos Raimundo Gomes de Freitas, Cláudio Tinoco Mesquita

**Affiliations:** Universidade Federal Fluminense, UFF, Niterói, RJ - Brazil

**Keywords:** Amyloidosis, Familial / complications, Cardiomyopathy, Restrictive / complications, Diagnostic Imaging, Echocardiography / methods, Prealbumin / analysis

## Abstract

**Background:**

Amyloidosis is a disease caused by deposits of insoluble fibrils in extracellular spaces. The most common type of familial amyloidosis is mediated by mutation of transthyretin, especially Val30Met. Symptoms and ejection fraction decrease may occur in cardiac amyloidosis only in case of poor prognosis. Myocardial strain detected by two-dimensional speckle tracking echocardiography can indicate changes in myocardial function at early stages of the disease.

**Objective:**

To determine the accuracy of left ventricular longitudinal strain by two-dimensional speckle tracking echocardiography in patients with familial amyloidosis caused by Val30Met transthyretin mutation.

**Methods:**

Eighteen consecutive patients, carriers of transthyretin mutation, were evaluated by two-dimensional speckle tracking echocardiography, by which myocardial strain curves were obtained, following the American Society of Echocardiography recommendations.

**Results:**

Patients were divided into three groups: 1- Val30Met with cardiac amyloidosis; 2-Val30Met with extracardiac amyloidosis; 3 - Val30Met without evidence of disease. As the three groups were compared by the Mann-Whitney test, we found a statistically significant difference between groups 1 and 2 in the mean longitudinal tension (p=0.01), mean basal longitudinal strain (p=0.014); in mean longitudinal tension and mean longitudinal strain between groups 1 and 3 (p=0.005); and in the ratio of longitudinal strain of apical septum segment to longitudinal strain of basal septum (p=0.041) between groups 2 and 3.

**Conclusion:**

Left ventricular longitudinal strain detected by two-dimensional speckle tracking echocardiography is able to diagnose left ventricular dysfunction in early stages of familial amyloidosis caused by transthyretin Val30Met mutation.

## Introduction

Amyloidosis is a rare disease caused by deposits of proteins in the extracellular space of organs and tissues. The familial forms of the disease are commonly associated with mutations of genes related to proteins. The most frequent is transthyretin (TTR), a protein that is synthesized in the liver, choroid plexus and retina, and that acts in the transport of thyroxine (T4) and retinol-binding protein in the blood.^1^ The TTR gene is located on chromosome 18q12.1.^2^ The best described, most prevalent mutation is Val30Met (a methionine substitution for valine at position 30), which predominantly affects patients from Japan, Portugal, Sweden and Brazil.^3 ^Amyloidosis symptoms appear in the third to fifth decades of life, including progressive polyneuropathy, postural hypotension, and mild myocardial infiltration. The main clinical manifestations of cardiac amyloidosis (CA) are: restrictive heart disease, systolic dysfunction, postural hypotension and conduction disturbances. Rapezzi et al. reported a 98% survival after two years of TTR CA.^4^

**Figure 1:**
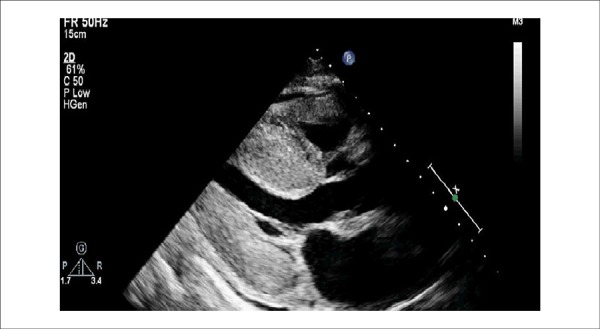
Echocardiography of cardiac amyloidosis with important left ventricular and right ventricular hypertrophy, increased thickness of mitral and aortic valves, and mild pericardial effusion.

Echocardiography is the cornerstone for evaluation of CA due to ease of image acquisition and interpretation, relative low cost, and capacity for unparalleled assessment of diastolic function and serial studies. The echocardiogram may show symmetrical thickening of left ventricular (LV) wall, hypokinesia, right ventricular free wall thickening, atrial septal thickening, valve thickening or valve failure, atrial dilatation and pericardial effusion^5^ (Figure 1). Two dimensional (2D) speckle tracking echocardiography (STE) consists in the capture and tracking of speckles along the cardiac cycle, generating motion vectors and deformation curves. The method has been used to measure myocardial deformation and the percentage of deformation. LV global systolic function remains normal until the final stages of CA. However, in contrast to LV ejection fraction (LVEF) and shortening fraction, the global longitudinal strain may be altered in the first stages of the disease. Thus, new imaging techniques, as the STE, have been suggested for the evaluation of patients with CA.^6-14^ The aim of our study was to determine the accuracy of LV longitudinal strain obtained by two-dimensional STE in a group of patient with familial amyloidosis caused by the Val30Met mutation of TTR.

## Methods

This is a cross-sectional, descriptive, comparative study on CA patients and genetic carriers of Val30Met mutation, and individuals without the mutation and without cardiovascular diseases as controls. Twenty-eight patients were selected and assessed from February 2014 to March 2015. Patients were divided into four groups - patients with familial CA with Val30Met mutation (n=6); patients with Val30Met mutation with diagnosis of familial extracardiac amyloidosis confirmed by biopsy (n=4); patients with Val30Met without amyloidosis (n=4); and a control group (n=14).

Patients were enrolled in the outpatient neurology service of Antonio Pedro University Hospital and in private offices of neurology run by professors of this hospital, and by contact to the Brazilian Association of Paramyloidosis by internet.

Sample calculation was based on the following assumptions:

In a similar study, Phelan et al.^15^ selected 26 patients with TTR. We took this number as a reference for our sample calculation.

The expected margin of error is 5%, with 95% confidence error. The distribution of CA in this population is unknown; however, we extrapolated the value of 50% of primary amyloidosis (AL) distribution, and used it as reference for our sample calculation.

We used the online sample calculator available at http://www.raosoft.com/samplesize.html, and the sample size calculated was 26.

### Inclusion criteria

Age > 18 years.

Agreement to participate and signature of the informed consent form

Patients carriers of TTR genetic mutation and/or with diagnosis of TTR amyloidosis (ATTR).

### Exclusion criteria

1. Poor quality of two-dimensional echocardiogram, established as the presence of artifacts or poor visualization of more than two cardiac segments.
2. Tachycardia (heart rate over 100 bpm).
3. Atrial fibrillation or other arrhythmias with variation in the R-R interval.
4. Other causes of ventricular hypertrophy - systemic arterial hypertension (SAH), hypertrophic cardiomyopathy (HCM), aortic stenosis, Fabry disease.

Patients attended a medical visit, during which their demographic data were collected, and anamnesis and physical exam were conducted. Electrocardiography (ECG), conventional echocardiography (echo) and two-dimensional STE were performed.

The echocardiographic images were obtained using a Philips IE33 equipment (Philips Medical Systems, Bothel, Washington, USA), with a 5-1 MHz Sector S5-1 transducer. The quantification of cardiac chambers, hemodynamic measurements, tissue Doppler study and two-dimensional RTE were performed offline, using Q-Lab 5.0 (Advanced Quantification Software - Philips Medical Systems, Bothell, WA, USA), a specific software for analysis of digital images, according to the protocols of the American Society of Echocardiography.^13,14,16,17^

Echocardiographic measurements were taken three times, and the mean of these values was recorded.

Numerical data were described by descriptive analysis in tables, and expressed as mean, standard deviation and median.

The Mann-Whitney test was used to assess differences in clinical variables of ECG, echo and two-dimensional STE between the paired groups. The normality of variable distribution was tested by the Shapiro-Wilk test. Significance level was set at 5%. Statistical analysis was processed with the SAS 6.11(SAS Institute, Inc., Cary, NC) software.

## Results

Tables 1, 2, and 3 present numerical clinical variables in mean, standard deviation and median of numerical clinical variables of ECG, echo and two-dimensional STE according to the pairs of groups G1 x G2, G1 x G3, and G1 x G4, and corresponding descriptive level (p-value) of Mann-Whitney test.

**Table 1:** Comparison of echocardiographic findings between group 1 (G1) and group 2 (G2)

Variable	G1: Cardiac amyloidosis					G2: Extracardiac amyloidosis					p value*
	n	mean	±	SD	med	n	mean	±	SD	med	
Left atrial diameter (cm)	6	4.00	±	0.18	4.00	4	3.09	±	0.42	3.20	0.009
LV end-systolic diameter (cm)	6	1.97	±	0.47	2.03	4	2.14	±	0.19	2.06	0.67
LV posterior wall (cm)	6	1.07	±	0.39	1.01	4	0.660	±	0.098	0.680	0.033
LV end-diastolic diameter (cm)	6	3.86	±	0.66	4.11	4	4.53	±	0.51	4.49	0.20
Interventricular septum (cm)	6	1.17	±	0.60	1.08	4	0.725	±	0.033	0.735	0.086
Mean wall thickness (cm)	6	1.12	±	0.49	1.03	4	0.690	±	0.067	0.705	0.055
Relative wall thickness	6	0.600	±	0.346	0.500	4	1.200	±	1.867	0.300	0.38
End-systolic volume -Teicholz (mL)	6	12.7	±	7.7	11.7	4	15.1	±	3.7	13.6	0.52
% shortening -Teicholz (%)	6	50.0	±	6.8	48.8	4	52.9	±	2.6	52.5	0.29
Ejection fraction - Teicholz (%)	6	81.5	±	6.5	81.1	4	84.0	±	1.9	83.6	0.39
Ejection fraction - Simpson bp (%)	6	77.2	±	12.9	80.5	4	73.3	±	3.7	73.8	0.29
LAV - apical 4 chambers (mL)	6	39.3	±	12.6	37.0	4	32.8	±	15.5	35.0	0.52
Indexed LAV - apical 4chambers (mlLm^2^)	6	23.9	±	7.1	25.0	4	18.6	±	8.1	19.8	0.20
LAV - apical 2 chambers (mL)	6	37.2	±	12.6	38.0	4	22.3	±	7.4	22.0	0.088
Indexed LAV - apical 2chambers (ml/m^2^)	6	23.1	±	9.5	23.2	4	13.2	±	5.3	12.6	0.055
LAV - biplane (ml)	6	38.7	±	10.7	41.5	4	26.4	±	7.2	27.0	0.088
Indexed LAV - biplane (mL/m^2^)	6	23.7	±	7.3	26.3	4	15.4	±	3.9	16.4	0.055
Medial E/E' ratio	5	13.4	±	5.5	12.5	3	8.2	±	1.8	9.0	0.10
Lateral E/E' ratio	5	10.7	±	7.9	7.5	4	6.4	±	0.1	6.4	0.14
Right ventricular lateral E'-wave velocity on tissue Doppler (cm/s)	5	11.1	±	1.1	11.6	1	11.7				0.55
Mean basal longitudinal strain(%)	6	-11.6	±	3.1	-12.0	4	-19.9	±	3.9	-20.5	0.014
Apical longitudinal strain- 2 chambers (%)	6	-16.0	±	3.9	-15.5	4	-23.0	±	1.4	-23.5	0.023
Apical longitudinal strain - 4 chambers (%)	6	-17.0	±	0.9	-17.0	4	-22.5	±	3.1	-22.5	0.009
Apical longitudinal strain.- apical longitudinal. (%)	6	-16.7	±	1.4	-16.5	4	-22.0	±	3.5	-23.0	0.030
Mean longitudinal tension - mean (%)	6	-16.8	±	1.8	-16.0	4	-22.3	±	1.3	-22.0	0.009

LV: left ventricular; LAV: left atrial volume; E/E' ratio: ratio between atrial flow E wave on Doppler and E' wave on tissue Doppler;  (*) Mann-Whitney test; values in mean ± standard deviation (SD) and median (med).

**Table 2:** Comparison of echocardiographic findings between group 1 (G1) and group 3 (G3)

Variable	G1: Cardiac amyloidosis					G3: TTR mutation without the disease					p value*
	n	mean	±	SD	med	n	mean	±	SD	med	
Left atrial diameter (cm)	6	4.00	±	0.18	4.00	4	3.15	±	0.35	3.15	0.009
LV end-systolic diameter (cm)	6	1.97	±	0.47	2.03	4	2.45	±	0.22	2.42	0.055
LV posterior wall (cm)	6	1.07	±	0.39	1.01	4	0.618	±	0.026	0.620	0.010
LV end-diastolic diameter (cm)	6	3.86	±	0.66	4.11	4	4.64	±	0.26	4.60	0.033
Interventricular septum (cm)	6	1.17	±	0.60	1.08	4	0.628	±	0.072	0.620	0.041
Mean wall thickness (cm)	6	1.12	±	0.49	1.03	4	0.623	±	0.048	0.620	0.024
Relative wall thickness	6	0.600	±	0.346	0.500	4	0.250	±	0.058	0.250	0.026
End-systolic volume -Teicholz (mL)	6	12.7	±	7.7	11.7	4	22.8	±	3.9	23.4	0.055
% shortening -Teicholz (%)	6	50.0	±	6.8	48.8	4	45.9	±	4.1	45.8	0.45
Ejection fraction - Teicholz (%)	6	81.5	±	6.5	81.1	4	77.1	±	4.3	77.0	0.39
Ejection fraction - Simpson bp (%)	6	77.2	±	12.9	80.5	4	74.7	±	7.9	75.2	0.45
LAV - apical 4 chambers (mL)	6	39.3	±	12.6	37.0	4	29.8	±	4.4	30.0	0.11
Indexed LAV - apical 4chambers (mlLm^2^)	6	23.9	±	7.1	25.0	4	17.4	±	2.3	16.9	0.088
LAV - apical 2 chambers (mL)	6	37.2	±	12.6	38.0	4	26.0	±	3.5	27.0	0.20
Indexed LAV - apical 2chambers (ml/m^2^)	6	23.1	±	9.5	23.2	4	15.0	±	1.1	15.4	0.087
LAV - biplane (ml)	6	38.7	±	10.7	41.5	4	27.1	±	4.2	26.7	0.14
Indexed LAV - biplane (mL/m^2^)	6	23.7	±	7.3	26.3	4	15.9	±	2.6	15.6	0.088
Medial E/E' ratio	5	13.4	±	5.5	12.5	4	8.1	±	1.9	8.6	0.086
Lateral E/E' ratio	5	10.7	±	7.9	7.5	4	5.0	±	0.3	5.0	0.14
Right ventricular lateral E'-wave velocity on tissue Doppler (cm/s)	5	11.1	±	1.1	11.6	4	13.1	±	1.2	13.2	0.036
Mean basal longitudinal strain(%)	6	-11.6	±	3.1	-12.0	4	-20.6	±	2.6	-20.7	0.010
Apical longitudinal strain- 2 chambers (%)	6	-16.0	±	3.9	-15.5	4	-22.0	±	1.8	-22.0	0.041
Apical longitudinal strain - 4 chambers (%)	6	-17.0	±	0.9	-17.0	4	-20.5	±	2.5	-20.0	0.016
Apical longitudinal strain.- apical longitudinal. (%)	6	-16.7	±	1.4	-16.5	4	-19.5	±	2.9	-19.5	0.10
Mean longitudinal tension - mean (%)	6	-16.8	±	1.8	-16.0	4	-20.5	±	0.6	-20.5	0.016

TTR: transthyretin LV: left ventricular; LAV: left atrial volume; E/E' ratio: ratio between atrial flow E wave on Doppler and E' wave on tissue Doppler; (*)Mann-Whitney test; values in mean ± standard deviation (SD) and median (med).

**Table 3:** Comparison of echocardiographic findings between group 1 (G1) and group 4 (G4)

Variable	G1: Cardiac amyloidosis					G4: control					p value*
	n	mean	±	SD	med	n	mean	±	SD	med	
Left atrial diameter (cm)	6	4.00	±	0.18	4.00	14	3.06	±	0.33	3.10	0.0005
LV end-systolic diameter (cm)	6	1.97	±	0.47	2.03	14	2.65	±	0.39	2.65	0.009
LV posterior wall (cm)	6	1.07	±	0.39	1.01	14	0.681	±	0.104	0.695	0.008
LV end-diastolic diameter (cm)	6	3.86	±	0.66	4.11	14	4.75	±	0.64	4.71	0.029
Interventricular septum (cm)	6	1.17	±	0.60	1.08	14	0.671	±	0.103	0.675	0.010
Mean wall thickness (cm)	6	1.12	±	0.49	1.03	14	0.674	±	0.080	0.653	0.011
Relative wall thickness	6	0.600	±	0.346	0.500	14	0.293	±	0.047	0.300	0.005
End-systolic volume -Teicholz (mL)	6	12.7	±	7.7	11.7	14	26.7		9.8	25.8	0.008
% shortening -Teicholz (%)	6	50.0	±	6.8	48.8	14	44.2		3.8	44.1	0.069
Ejection fraction - Teicholz (%)	6	81.5	±	6.5	81.1	14	75.2		4.2	75.4	0.032
Ejection fraction - Simpson bp (%)	6	77.2	±	12.9	80.5	14	71.7		6.2	70.5	0.14
LAV - apical 4 chambers (mL)	6	39.3	±	12.6	37.0	14	26.4		9.4	28.5	0.028
Indexed LAV - apical 4chambers (mlLm^2^)	6	23.9	±	7.1	25.0	14	14.7		5.2	14.5	0.010
LAV - apical 2 chambers (mL)	6	37.2	±	12.6	38.0	14	29.1		12.9	26.0	0.11
Indexed LAV - apical 2chambers (ml/m^2^)	6	23.1	±	9.5	23.2	14	15.6		5.2	13.3	0.083
LAV - biplane (ml)	6	38.7	±	10.7	41.5	14	28.3		9.4	28.0	0.063
Indexed LAV - biplane (mL/m^2^)	6	23.7	±	7.3	26.3	14	15.3		4.1	16.1	0.026
Medial E/E' ratio	5	13.4	±	5.5	12.5	14	6.9		1.7	6.6	0.005
Lateral E/E' ratio	5	10.7	±	7.9	7.5	14	4.9		0.9	4.6	0.033
Right ventricular lateral E'-wave velocity on tissue Doppler (cm/s)	5	11.1	±	1.1	11.6	12	14.2		2.6	13.1	0.003
Mean basal longitudinal strain(%)	6	-11.6	±	3.1	-12.0	14	-21.2	±	3.2	-21.0	0.0005
Apical longitudinal strain- 2 chambers (%)	6	-16.0	±	3.9	-15.5	14	-19.2	±	3.3	-17.5	0.015
Apical longitudinal strain - 4 chambers (%)	6	-17.0	±	0.9	-17.0	14	-19.0	±	2.2	-19.5	0.054
Apical longitudinal strain.- apical longitudinal. (%)	6	-16.7	±	1.4	-16.5	14	-17.7	±	2.3	-18.0	0.26
Mean longitudinal tension - mean (%)	6	-16.8	±	1.8	-16.0	14	-18.6	±	2.3	-18.0	0.077

LV: left ventricular; LAV: left atrial volume; E/E' ratio: ratio between atrial flow E wave on Doppler and E' wave on tissue Doppler; (*)Mann-Whitney test; values in mean ± standard deviation (SD) and median (med).

**Graph 1:**
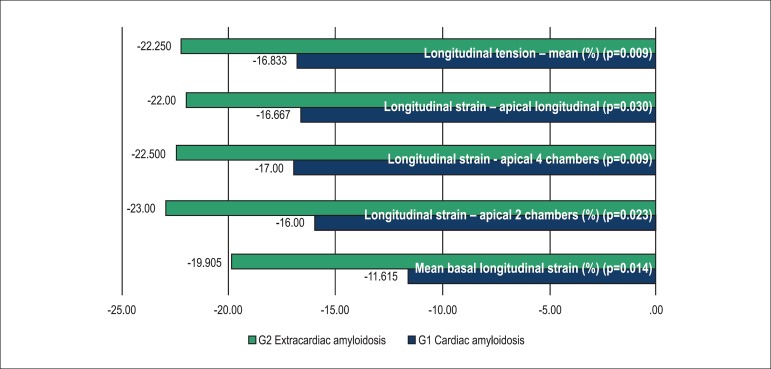
Comparison of longitudinal strain between group 1 (G1) and group 2 (G2)

**Graph 2:**
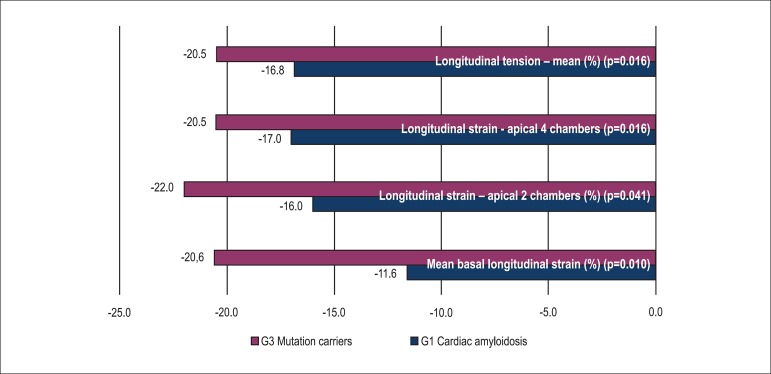
Comparison of longitudinal strain between group 1 (G1) and group 3 (G3)

**Graph 3:**
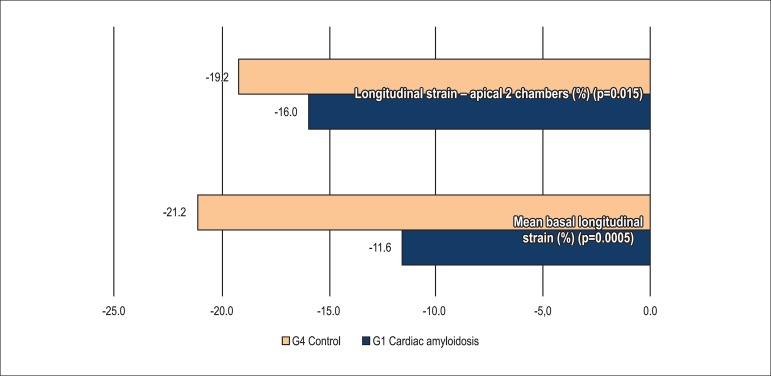
Comparison of longitudinal strain between group 1 (G1) and group 4 (G4)

Group 1 corresponds to patients with familial CA, according to the international criterion for the disease - mean LV wall thickness ≥ 12 mm, diastolic dysfunction ≥ stage 2, or global longitudinal strain lower than -18%. Group 2 corresponds to patients with familial extracardiac amyloidosis confirmed by biopsy. Group 3 corresponds to patients with TTR mutation with no evidence of CA. Group 4 is the control group (see Tables and Graphs 1, 2 and 3).

## Discussion

As we compared the mean values of longitudinal strain per LV segments between G1 and G3, we found a statistically significant decrease in these values in the basal, anterolateral (p=0.019) and in the medial inferolateral segments in G1 (p=0.042). The same was observed in the basal inferoseptal (p=0.009), anteromedial (p=0.010), inferior medial (p=0.054), inferolateral medial (p=0.010) and apical septum (p=0.032) in G2, and in the basal inferoseptal (p=0.006), basal anterior (p=0.017), basal inferior (p=0.031), basal anterolateral (p=0.001), basal inferolateral (p=0.010), medial inferior (p=0.025) and apical anterior (p=0.013) in G4. It is of note that mean values of longitudinal strain were decreased in G1 not only as compared with G3, but also in absolute values (<-18%). On the other hand, longitudinal strain of apical segments, even when decreased in relation to other groups, was not decreased in absolute values.

**Figure 2:**
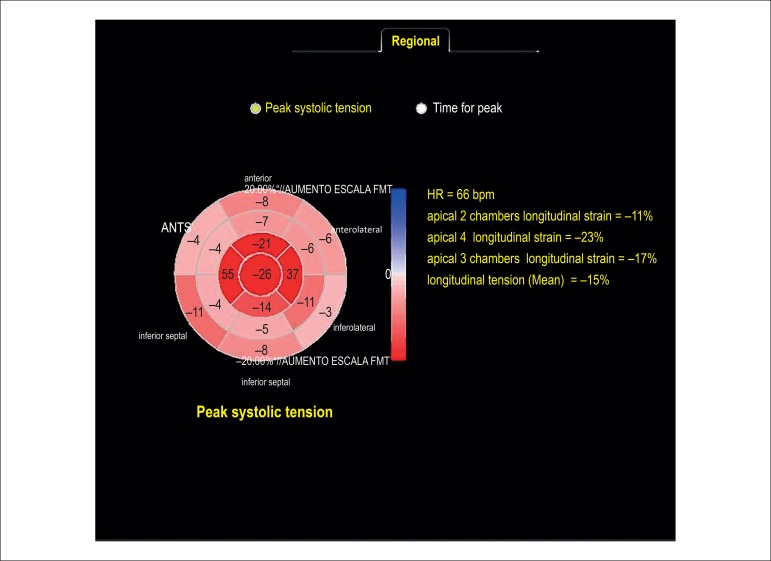
Bull's-Eye graph of cardiac amyloidosis patient. Apical longitudinal strain is preserved and there is important decrease in basal and medial segments.

The two-dimensional STE showed that apical segments were not affected by amyloidosis, ATTR or AL, which differs from the pattern of both HCM and aortic stenosis that do not spare the apex. Only apical strain was different between ATTR and AL, and significantly lower in ATTR patients. However, no difference was detected in global longitudinal strain or in the mean basal or medial longitudinal strain between the two types of amyloidosis^15^ (see Figure 2).

Mean basal longitudinal strain was lower in G1 as compared with G3 (p=0.010), G2 (p=0.014) and G4 (p=0.0005). According to Baccouche et al. this parameter has a differential diagnostic value between AC and HCM.^18^

When we evaluated global longitudinal strain in G1, the mean longitudinal strain in the apical two-chamber, four-chamber and three-chamber views (longitudinal apical), as well as the mean longitudinal tension were significantly lower than in G2, G3 and G4 groups.

Serial analysis showed that systolic strain rate and strain at the basal and mid ventricle were significantly reduced in asymptomatic patients with increased LV wall thickness.^13^ In addition, the longitudinal systolic rate was reduced in all 16 segments of left ventricle in CA patients, who did not have abnormal echo findings.^19^

There are few studies showing functional changes in patients with CA using two-dimensional STE. Sun et al. showed that global longitudinal strain detected by two-dimensional STE was significantly lower (12%) in CA patients as compared with healthy controls, and also compared with patients with LV hypertrophy caused by HCM or hypertensive disease.^20^

Liu et al. showed that the ejection fraction was preserved whereas longitudinal strain was notably reduced in both compensated and decompensated patients (New York Heart Association functional class > 2).^21^

Disturbances in electrical conduction are among the main clinical manifestations of CA, and may be present in up to one third of patients.^5^ Patients in G1 had increased PR interval (mean of 0.230s ± 0.060), which was significantly different from G2 (p=0.015), G3 (p=0.044) and G4 (p=0.005). Sayed et al.^22^ also showed an association between disturbances in electrical conduction and decreased longitudinal strain, and a negative prognostic value for this association, although the study was conducted with cardiac AL amyloidosis.

Left atrial diameter was significantly greater in the G1 group (4.0 cm ± 0.18) than in G3 (p = 0.009), G2 (p = 0.009) and G4 (p= 0.0005). We also found increased left atrial volume in G1 in the apical four-chamber view (p=0.028), biplane (p=0.026) and indexed left atrial volume in the apical four-chamber view (p=0.010) as compared with G4. Such difference may be a sign of increased pressure in the left ventricle due to restrictive diastolic function, leading to pressure overload in the left atrium.^23^

Mean values of LV end-systolic diameter and end-systolic volume were significantly lower in G1 (p=0.009 and p=0.008, respectively) and G2 (p=0.025 and p=0.025, respectively) as compared with G4. LV end-systolic volume was also significantly lower (p=0.043) in G2 than G4. G1 showed mean LV end-diastolic diameter and end-diastolic volume significantly lower than G3 (p=0.033 and p=0.033, respectively), and G4 (p=0.029 and p=0.028, respectively). CA progresses with mild decrease in LV cavity.^24,25^

Variables related to hypertrophy caused by amyloid deposit were statistically different between G1 and G3. Mean diastolic thickness of the interventricular septum in G1 was increased (1.17 ± 0.60 cm), and statistically greater than G3 (p=0.041) and G4 (p=0.010). The same was observed with LV posterior wall end-diastolic thickness, with mean of 1.07 ± 0.39 cm in G1, which was significantly different as compared with G3 (p=0.010), G2 (p=0.033) and G4 (0.008). Mean values of interventricular septum was greater in G1 (1.17±0.60 cm) than in G3 (p=0.041) and G4 (p=0.010). Mean wall thickness was significantly greater in G1 than in G3 (p=0.024) and G4 (p=0.011). Mean relative wall thickness was also increased in G1 (0.600 ± 0.346) compared with G3 (p=0.026) and G4 (p=0.005). Our study shows a relationship between LV wall hypertrophy and severity of disease. Mean wall hypertrophy greater than 15mm has been shown as an independent negative prognostic factor.^26^

Amyloid deposits cause restrictive heart disease. The pattern of echocardiographic parameters of diastolic dysfunction classification tend to worsen with the disease progression.^23^ Patients of G2 also showed increased mean lateral E/E' ratio compared with G3 (p=0.020). Lateral E'-wave velocity on tissue Doppler was also decreased in G2 as compared with G3 (p=0.021). Medial and lateral E/E' ratio in G1 were increased (p=0.005 and p=0.033, respectively) compared with G4.

Tricuspid annular S'-wave velocity on tissue Doppler is an index of right ventricular systolic function, with normal values greater than 10 cm/s. Its mean values in G1 was significantly different from G3 (p=0.036) and G4 (p=0003). Capelli et al.^27^ showed statistically significant difference in tricuspid annulus tissue Doppler analysis between patients with CA, patients with extracardiac amyloidosis and healthy controls, and reported a negative prognostic value for right ventricular systolic dysfunction.

## Conclusions

Two-dimensional STE increased the sensitivity of echo in diagnosing CA caused by TTR Val30Met mutation, since the adoption of a global longitudinal strain < -18% criterion increased the number of diagnosed patients from two (diagnosed by echo) to six patients.
